# Early-onset mild cognitive impairment in Parkinson’s disease: Altered corticopetal cholinergic network

**DOI:** 10.1038/s41598-017-02420-w

**Published:** 2017-05-24

**Authors:** Injoong Kim, Na-Young Shin, Yunjin Bak, Phil Hyu Lee, Seung-Koo Lee, Soo Mee Lim

**Affiliations:** 1 Department of Radiology, Veterans Health Service Medical Center, Seoul, 05368 Korea; 2 Department of Radiology, College of Medicine, The Catholic University of Korea, Seoul, 06591 Korea; 3 Department of Neurology, Yonsei University College of Medicine, Seoul, 03722 Korea; 4 Department of Radiology, Yonsei University College of Medicine, Seoul, 03722 Korea; 5 Department of Radiology, Ewha Womans University School of Medicine, Seoul, 07985 Korea

## Abstract

Degeneration of the substantia innominata (SI) is significantly correlated with cognitive performance in Parkinson’s disease (PD). We examined functional and structural patterns of SI degeneration in drug-naïve PD patients according to the duration of parkinsonism before mild cognitive impairment (MCI) diagnosis. Twenty PD patients with a shorter duration (PD-MCI-SD, <1 year), 18 patients with a longer duration (PD-MCI-LD, ≥1 year), and 29 patients with intact cognition (PD-IC) were included. Seed-based resting-state functional connectivity (rsFC) analysis using bilateral SI seed and region-of-interest-based volumetric analysis were performed. Compared to PD-IC, the collapsed PD-MCI group showed altered rsFC in the right frontal and bilateral parietal areas. PD-MCI-SD showed rsFC alteration in broader frontal and parietal areas compared to the other groups. Decreased rsFC in the right frontal area was also significantly correlated with shorter disease duration. No significant SI volume change was found between the groups. Altered rsFC between the SI and the frontal and parietal areas might be relevant to cognitive dysfunction in PD. Decreased rsFC between the SI and frontal area might be associated with early-onset MCI, suggesting that cholinergic deficits in the frontal brain areas might play an important role in the acceleration of cognitive decline in PD.

## Introduction

Parkinson’s disease (PD) is a neurodegenerative disease mainly characterized by motor symptoms^1^. However, a substantial percentage of PD patients have non-motor symptoms as well. Cognitive dysfunction is a common non-motor symptom observed in PD patients and its severity varies from mild cognitive impairment (PD-MCI) to dementia (PDD)^2, 3^.

Although the neural basis for cognitive dysfunction in PD remains unknown, pathological and neuroimaging studies suggest that the cholinergic system arising from the nucleus basalis of Meynert (NBM) located in the substantia innominata (SI) of the basal forebrain plays an important role in the cognitive functions of PD patients. Cortical cholinergic deficits resulting from NBM neuronal loss have been strongly correlated with cognitive impairment in a past study^4^. Also, a previous study using structural magnetic resonance imaging (MRI) demonstrated that the SI volume in PD differs depending on cognitive status and that the SI volume is significantly correlated with cognitive performance^5^. Previous positron emission tomography (PET) studies using *in vivo* imaging of cerebral acetylcholinesterase have also demonstrated that cholinergic dysfunction occurs even in the early course of PD and that it is more widespread and profound in PDD^6, 7^.

Patients with early-onset of PDD are reported to have higher pathological burden^8, 9^. Therefore, we postulated that PD patients with early-onset of MCI might also show more pathological burden on the cholinergic system of the SI than those with late-onset of MCI. This different underlying neuropathology may influence both the functional and structural patterns of SI degeneration. Given that cholinergic projections from the SI innervate the entire cerebral cortex, we need to identify the specific brain cortical regions relevant to early-onset MCI, in other words, rapid cognitive decline, to understand the underlying pathophysiology of SI degeneration and to define new treatment targets.

Resting-state functional connectivity (rsFC) can be used to evaluate altered relationships between the SI and particular areas of the whole brain, which means it can be used to define brain regions relevant to rapid cognitive decline. We also measured the SI volume to see whether structural degeneration was associated with the cognitive decline rate. Accordingly, we aimed to define different functional as well as structural patterns of SI degeneration in drug-naïve PD patients according to the duration of parkinsonism before MCI diagnosis using resting-state functional MRI (rsfMRI) and region-of-interest (ROI)-based volumetric analyses.

## Results

### Demographic and clinical characteristics

Among the 239 PD patients who underwent both MRI and neuropsychological tests, 38 age-, sex-, and years of education-matched drug-naïve patients were further classified into 20 patients with a shorter duration of parkinsonism before MCI diagnosis (the PD-MCI-SD group, <1 year) and 18 patients with a longer duration (the PD-MCI-LD group, ≥1 year), respectively. Twenty-nine drug-naïve PD patients with intact cognition were also included for comparison (the PD-IC group). Demographic and clinical data of the patients are summarized in Table 1. The median duration of parkinsonism was 6.0 (range, 2–11) months in the PD-MCI-SD group and 25.0 (range, 12–85) months in the PD-MCI-LD group. The PD-MCI-LD group (29.0 ± 7.4) had a higher score in UPDRS-III, in other words, severer motor symptoms, compared to the PD-IC (18.9 ± 8.4; *P* < 0.001) and PD-MCI-SD groups (20.4 ± 6.7; *P* = 0.003). Both PD-MCI-SD (26.9 ± 1.9; *P* = 0.015) and PD-MCI-LD (26.2 ± 2.8; *P* = 0.009) groups had lower K-MMSE scores compared to the PD-IC group (28.3 ± 1.9). Other demographic and clinical characteristics were not significantly different between the groups.

**Table 1:** Demographic and clinical characteristics of the PD-IC group and PD-MCI groups according to the duration of parkinsonism before MCI diagnosis.

	PD-IC (n = 29)	PD-MCI-SD (n = 20)	PD-MCI-LD (n = 18)	*P* value^§^	Post-hoc analysis		
					*P* ^†^	*P* ^‡^	*P* ^¶^
Age, year	65.5 ± 9.4	67.0 ± 8.4	69.2 ± 7.4	0.373	0.824	0.339	0.719
Age at onset, year	64.2 ± 9.6	66.4 ± 8.4	66.4 ± 7.8	0.597	0.670	0.676	1.000
Male, n (%)	12 (41.3)	6 (30)	9 (50)	0.449	1.000	1.000	0.624
Education duration, year	10.7 ± 4.5	10.5 ± 5.5	7.8 ± 4.8	0.126	0.990	0.133	0.220
Parkinsonism duration prior to MCI, month, median (range)	NA	6 (2–11)	25 (12–85)				<0.001
UPDRS III	18.9 ± 8.4	20.4 ± 6.7	29.0 ± 7.4	<0.001	0.794	<0.001	0.003
K-MMSE	28.3 ± 1.9	26.9 ± 1.9	26.2 ± 2.8	0.002	0.015	0.009	1.000
BDI	11.0 ± 9.1	12.4 ± 7.1	9.7 ± 9.3	0.623	0.850	0.858	0.595
Interval between MR scan and NP test, d, median (range)	1.0 (0–125)	0 (0–35)	0 (0–43)	0.059	0.060	0.807	0.642

Abbreviations: BDI = Beck Depression Inventory; K-MMSE = the Korean version of the Mini Mental State Examination; MCI = mild cognitive impairment; n = number; NP test = neuropsychological test; PD-IC = Parkinson’s disease with intact cognition; PD-MCI-SD = Parkinson’s disease with a shorter duration of parkinsonism before mild cognitive impairment diagnosis; PD-MCI-LD = Parkinson’s disease with a longer duration of parkinsonism before mild cognitive impairment diagnosis; UPDRS III = Unified Parkinson’s Disease Rating Scale Part III.

Note.-Unless otherwise indicated, data are means ± standard deviations.

^§^ *P* values for comparison among the 3 groups; ^†^ *P* values for comparison between the PD-IC and PD-MCI-SD groups; ^‡^ *P* values for comparison between the PD-IC and PD-MCI-LD groups; ^¶^ *P* values for comparison between the PD-MCI-SD and PD-MCI-LD groups.

### Group comparisons of Neuropsychological data

Neuropsychological data are summarized in Table 2. Compared to the PD-IC group, the PD-MCI groups showed lower performances on most neuropsychological tests, but there was no significant difference between the PD-MCI-SD and PD-MCI-LD groups for neuropsychological performances.

**Table 2:** Neuropsychological data in the PD-IC group and PD-MCI groups according to the duration of parkinsonism before MCI diagnosis.

Cognitive Subdomains	PD-IC (n = 29)	PD-MCI-SD (n = 20)	PD-MCI-LD (n = 18)	*P* value^§^	Post-hoc analysis		
					*P* ^†^	*P* ^‡^	*P* ^¶^
Attention							
Digit Span (forward)	7 (4–9)	6 (4–8)	6 (3–8)	0.015	0.036	0.069	1.000
Digit Span (backward)	4 (3–8)	3 (2–5)	3 (0–6)	0.002	0.003	0.060−	1.000
Digit Span total	11.1 ± 2.2	9.3 ± 1.5	9.2 ± 2.5	0.003	0.011	0.010	0.983
Word Stroop test	112 (109–112)	112 (96–112)	112 (94–112)	0.041	0.033	0.855	0.768
Color Stroop test	91.8 ± 19.4	71.3 ± 23.4	72.0 ± 22.8	0.002	0.005	0.014	0.994
Executive function							
Phonemic generative naming	27 (9–44)	15.5 (8–47)	18 (3–37)	0.001	0.002	0.006	0.996
COWAT (animal)	16.7 ± 4.2	14.3 ± 2.7	14.5 ± 4.5	0.056	0.082	0.150	0.984
COWAT (supermarket)	17.8 ± 5.3	14.9 ± 5.5	13.8 ± 5.9	0.047	0.180	0.057	0.827
Clock Drawing test	10 (8–10)	10 (4–10)	9 (3–10)	0.154	—	—	—
Verbal memory function							
SVLT							
Free recall	22.2 ± 4.5	17.6 ± 4.4	17.3 ± 5.5	0.001	0.004	0.004	0.979
Delayed recall	7.3 ± 2.3	4.6 ± 2.5	4.6 ± 2.5	<0.001	0.001	0.001	1.000
Recognition	21.3 ± 1.9	19.9 ± 2.6	20.3 ± 2.0	0.098	—	—	—
Visual memory function							
RCFT immediate recall	18.8 ± 7.1	11.0 ± 6.4	11.0 ± 8.3	<0.001	0.001	0.002	1.000
RCFT delayed recall	18.4 ± 6.3	11.5 ± 6.7	11.4 ± 7.8	0.001	0.003	0.004	0.998
RCFT recognition	9.9 ± 1.8	9.0 ± 2.4	9.3 ± 2.2	0.319	0.303	0.654	0.874
Visuospatial function							
RCFT copy	34.0 ± 2.3	29.5 ± 6.7	29.1 ± 7.2	0.001	<0.001	<0.001	1.000
Pentagon drawing test	1 (0–1)	1 (0–1)	1 (0–1)	0.105	—	—	—
Language and related function							
K-BNT	49.7 ± 5.3	43.0 ± 8.0	43.8 ± 9.3	0.003	0.006	0.026	0.939
Other indices							
Contrasting program	20 (17–20)	20 (17–20)	20 (16–20)	0.140	—	—	—
Go-No-Go test	20 (10–20)	20 (3–20)	20 (6–20)	0.220	—	—	—
Semantic generative naming	33 (20–59)	27.5 (19–45)	27 (10–47)	0.028	0.083	0.050	0.950

Abbreviations: MCI = mild cognitive impairment; n = number; PD-IC = Parkinson’s disease with intact cognition; PD-MCI-SD = Parkinson’s disease with a shorter duration of parkinsonism before mild cognitive impairment diagnosis; PD-MCI-LD = Parkinson’s disease with a longer duration of parkinsonism before mild cognitive impairment diagnosis; COWAT = Controlled Oral Word Association Test; SVLT = Seoul Verbal Learning Test; RCFT = Rey complex figure test; K-BNT = Korean version of the Boston Naming Test.

Note.-Unless otherwise indicated, data are means ± standard deviations.

^§^ *P* values for comparison among the 3 groups; ^†^ *P* values for comparison between the PD-IC and PD-MCI-SD groups; ^‡^ *P* values for comparison between the PD-IC and PD-MCI-LD groups; ^¶^ *P* values for comparison between the PD-MCI-SD and PD-MCI-LD groups.

### Group comparisons of SI volume

There was no significant difference in SI volume between the collapsed PD-MCI group (0.977 ± 0.184 ml) and the PD-IC group (1.016 ± 0.147 ml; *P* = 0.359) as well as between all groups (PD-MCI-SD group, 0.971 ± 0.187 ml; PD-MCI-LD group, 0.984 ± 0.185 ml; and PD-IC group, 1.016 ± 0.147 ml; *P* = 0.641).

### Group comparisons of rsFC using the bilateral SI as the seed

**Figure 1:**
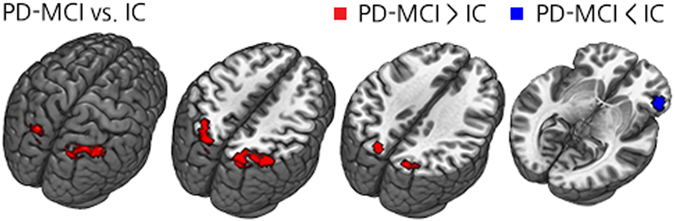
Group comparison of rsFC using the bilateral SI as the seed between the collapsed PD-MCI group and PD-IC group. Abbreviations: PD-MCI = Parkinson’s disease with mild cognitive impairment; PD-IC = Parkinson’s disease with intact cognition; rsFC =resting-state functional connectivity; SI = Substantia innominata.

Compared to the PD-IC group, the collapsed PD-MCI group showed decreased rsFC in the right frontal area, while showing increased rsFC in the bilateral parietal and occipital areas (Fig. 1 and Supplementary Table 1). Decreased rsFC in the right frontal area was correlated with attention and verbal memory function. Increased rsFC in the bilateral posterior brain areas was significantly correlated with decreased attention, executive function, visuospatial and visual memory function (Table 3).

**Table 3:** Correlation analysis between neuropsychological data and brain regions showing altered rsFC between the collapsed PD-MCI and PD-IC groups.

Cognitive Subdomains	Bilateral parietal areas		Right frontal area	
	*correlation coefficient*	*P value*	*correlation coefficient*	*P* value
Attention				
Digit Span (forward)	−0.151	0.228	0.112	0.372
Digit Span (backward)	−0.312	0.011	0.169	0.175
Digit Span total	−0.227	0.067	0.117	0.350
Word Stroop test	−0.188	0.138	0.240	0.056
Color Stroop test	−0.408*	0.001	0.330*	0.008
Executive function				
Phonemic generative naming	−0.425*	0.000	0.137*	0.275
COWAT (animal)	−0.157	0.209	0.184	0.139
COWAT (supermarket)	−0.431	0.000	0.180	0.148
Verbal memory function				
Free recall	−0.180	0.148	0.261	0.034
Delayed recall	−0.157*	0.207	0.254*	0.039
Recognition	−0.071	0.571	0.219	0.078
Visual memory function				
RCFT immediate recall	−0.245	0.047	0.120	0339
RCFT delayed recall	−0.145	0.244	0.076	0.544
RCFT recognition	−0.128*	0.306	−0.097*	0.438
Visuospatial function				
RCFT copy	−0.265*	0.032	0.079*	0.527
Pentagon drawing test	−0.123	0.326	0.110	0.380
Language and related function				
K-BNT	−0.144	0.247	0.163	0.192
Other indices				
Contrasting program	−0.169	0.175	0.275	0.026
Go-No-Go test	−0.269	0.029	0.228	0.066
Semantic generative naming	−0.330*	0.007	0.129*	0.302

^*^Pearson’s rho (*ρ*).

Unless otherwise indicated, data are Spearman’s *r*.

**Figure 2:**
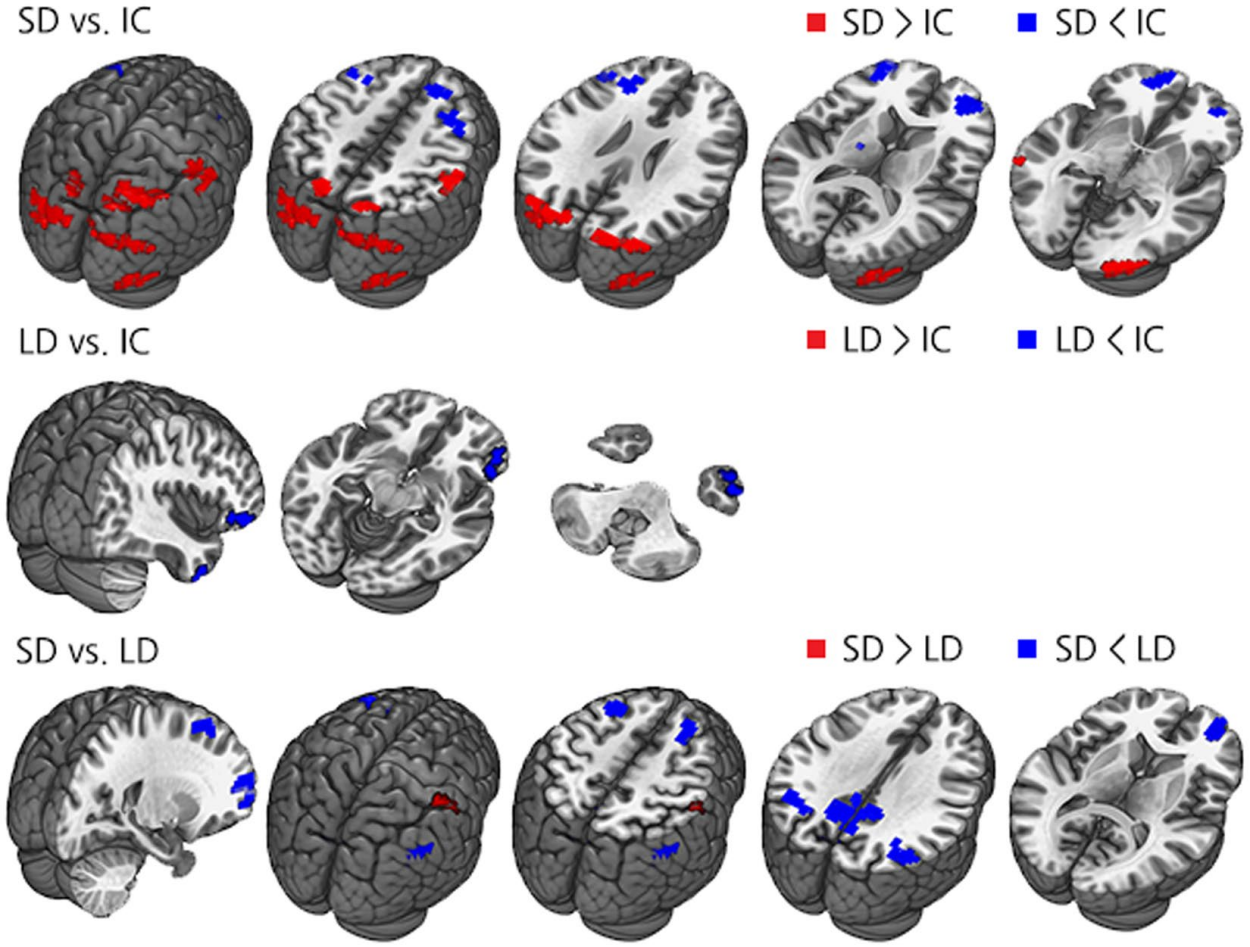
Group comparisons of rsFC using the bilateral SI as the seed in all group contrasts. Abbreviations: PD-MCI-SD = Parkinson’s disease with a shorter duration of parkinsonism before mild cognitive impairment diagnosis; PD-MCI-LD = Parkinson’s disease with a longer duration of parkinsonism before mild cognitive impairment diagnosis; PD-IC = Parkinson’s disease with intact cognition; rsFC = resting-state functional connectivity; SI = Substantia innominata.

Compared to the PD-IC group, the PD-MCI-LD group exhibited decreased rsFC in a few small areas involving the right inferior orbitofrontal and right inferior temporal gyri while no significantly increased rsFC was observed. On the other hand, the PD-MCI-SD group exhibited substantial altered rsFC relative to the PD-IC group with decreased rsFC being observed in the bilateral frontal areas; meanwhile, increased rsFC was observed in the bilateral parietal and occipital areas (Fig. 2 and Supplementary Table 1). Increased rsFC in the posterior cortical areas was attributable to loss of anti-correlation, while decreased rsFC in the frontal areas was attributable to declined positive correlation noted in the PD-IC group (Supplementary Figure 1).

When the PD-MCI-SD and PD-MCI-LD groups were directly compared, the PD-MCI-SD group showed decreased rsFC in the bilateral frontal and parietal areas including the precuneus, while increased functional connectivity was observed in the right primary somatosensory area (Fig. 2 and Supplementary Table 1). Significant group differences in the bilateral parietal areas and precuneus were attributable to the opposite direction of rsFC with the bilateral SI seed between the PD-MCI groups: positive correlation in the PD-MCI-LD group and negative correlation in the PD-MCI-SD group. On the other hand, decreased rsFC in the bilateral frontal areas was due to prominent anti-correlation between the bilateral SI seed in the PD-MCI-SD group (Supplementary Figure 2).

**Figure 3:**
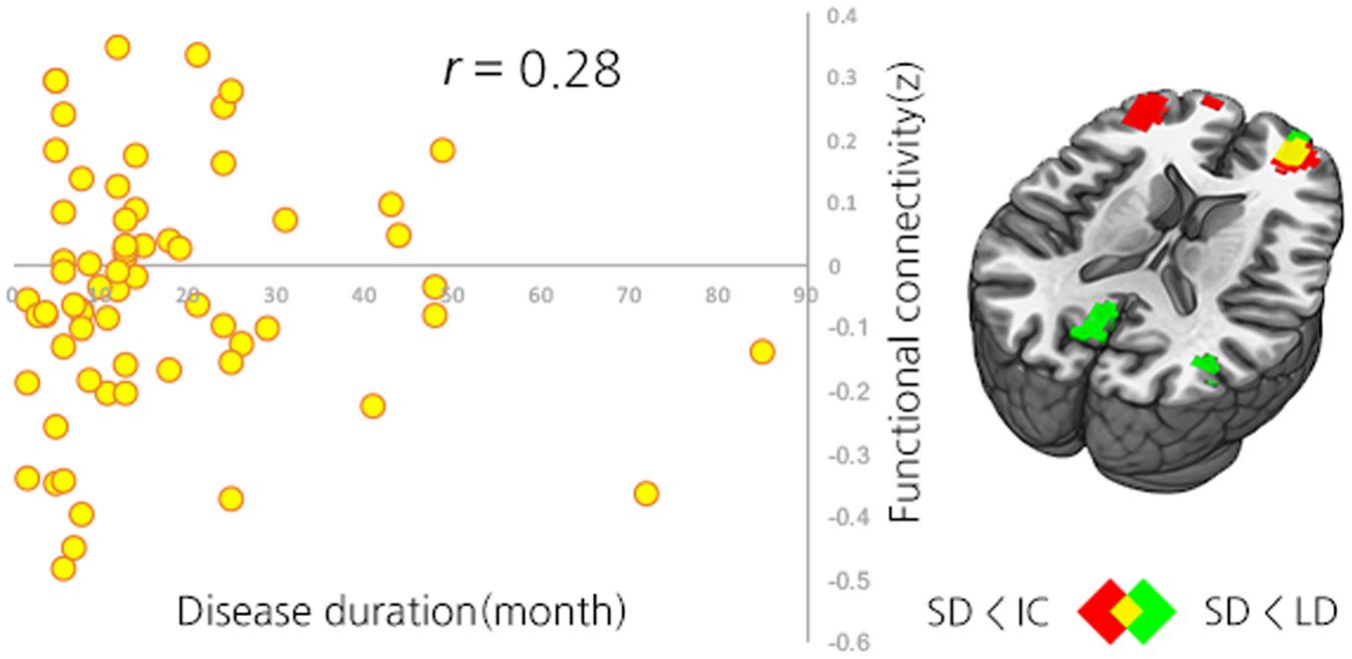
Correlation graph between the duration of parkinsonism before MCI diagnosis and rsFC between the bilateral SI seed and conjunction mask of the bilateral frontal areas (*r* = 0.28, *P* = 0.023). Abbreviations: PD-MCI-SD = Parkinson’s disease with a shorter duration of parkinsonism before mild cognitive impairment diagnosis; PD-MCI-LD = Parkinson’s disease with with a longer duration of parkinsonism before mild cognitive impairment diagnosis; PD-IC = Parkinson’s disease with intact cognition; rsFC = resting-state functional connectivity; SI = Substantia innominata.

To define brain regions relevant to disease duration, Spearman’s correlation analysis was performed between disease duration and rsFC in areas which showed significant group differences in PD-MCI-SD compared to both PD-MCI-LD and PD-IC. Among the three overlapped areas, only the right anterior frontal area (Montreal Neurological Institute [MNI] coordinates [x = 24, y = 57, z = 6]) showed significant associations between decreased rsFC and shorter disease duration (*ρ* = 0.277, *P* = 0.023; Fig. 3).

## Discussion

In this study, there was no significant structural change in SI between the groups; however, we found altered rsFC in the corticopetal cholinergic network according to not only the cognitive status but also duration of parkinsonism before MCI diagnosis in PD patients. There were three main findings in this study. First, decreased rsFC in the frontal areas and increased rsFC in the parietal areas with SI seed were associated with cognitive decline in drug-naïve PD patients. Second, patients with early-onset of MCI had prominent rsFC changes in these cognitive dysfunction-related areas, while little change was found in patients with late-onset of MCI. Lastly, decreased rsFC in the frontal area was associated with shorter disease duration before MCI diagnosis, suggesting that this area possibly plays a role in increasing the rate of cognitive decline.

In the present study, altered rsFC between the frontal and parietal areas with the bilateral SI seed was related with cognitive decline in PD patients. This result corresponds with previous imaging studies^10, 11^, which demonstrated a correlation between cognitive decline and progressive degeneration in the frontal and parietal cortices. Although frontal lobe dysfunction in PD is usually thought to be a result of the disturbed dopaminergic fronto-striatal network^12^, cholinergic dysfunction also contributes to frontal dysfunction. Previous studies have reported that cholinergic hypometabolism in the frontal cortex was greater in PDD compared to non-demented PD and decreased cholinergic function showed decreased executive and attentional function^13–15^. So, it is not surprising that we found decreased rsFC between the frontal areas and bilateral SI in this study. We also found an association between decreased rsFC in the right frontal area with attention and verbal memory dysfunction.

Involvement of the posterior brain areas in cognitive dysfunction has also been well reported in other studies on PD^16–18^. A previous study showed posterior cortical atrophy in the dorsal parietal as well as the occipitotemporal cortices of PDD patients^19^, and these areas overlapped a great deal with the areas showing cholinergic hypometabolism in those patients^20^. Baggio *et al*. also reported posterior brain atrophy in PD-MCI patients, but increased rsFC in the areas with the default mode network (DMN), which was correlated with visuospatial function^21^. The present study also showed increased rsFC in the bilateral parietal areas which was linked with cognitive dysfunction in PD patients, in contrary to the decreased rsFC observed in the frontal areas. Accordingly, we have to consider possible associations between cholinergic dysfunction, posterior cortical atrophy, and increased rsFC in this area. However, it remains unclear what causes these opposite patterns of rsFC between the frontal and parietal areas. According to a previous study with 225 normal subjects^22^, SI normally shows positive correlation with the frontal areas and negative correlation with the posterior brain areas. Our results also showed that decreased frontal connectivity was attributable to loss of positive correlation observed in the PD-IC group, and increased posterior brain connectivity to loss of negative correlation. Therefore, rsFC phenotypes caused by cholinergic deficits might seem to have opposite patterns depending on the inherent characteristics of the brain regions.

As we mentioned above, cognitive dysfunction-related changes in rsFC were prominent in the PD-MCI-SD group, but not in the PD-MCI-LD group when compared to the PD-IC group. These results suggest that the more disturbed intrinsic corticopetal cholinergic network might cause earlier-onset of MCI in the PD-MCI-SD group. In contrast, the PD-MCI-LD group with only minimal alteration in the cholinergic network was thought to preserve cognitive function for a relatively long disease duration, supporting our assumption that the PD-MCI-SD group has more burden in at least the corticopetal cholinergic system. This also corresponds to the ‘dual-syndrome hypothesis’ of cognitive impairment in PD suggested by Kehagia *et al*.^23^. Kehagia *et al*. proposed that while cognitive deficits in early PD are mainly mediated by dysfunction in the fronto-striatal dopaminergic network, progressive cognitive decline leading to dementia in PD is associated with superimposition of additional basal forebrain cholinergic system degeneration. However, other than dopaminergic and cholinergic denervation, depletion of other various neurotransmitters and genotypes is also known to contribute to cognitive dysfunction in PD^24, 25^. Therefore, future studies are needed to understand how these factors affect the rate of cognitive decline in PD patients.

We also found decreased rsFC between the right anterior frontal area and bilateral SI seed and this was associated with a shorter duration of parkinsonism before MCI diagnosis, a possible sign of rapid cognitive decline, in PD patients. Previous longitudinal studies have shown that impairments of posterior cortical function are predictors for future dementia in PD^16–18^. However, there is still debate on the prognostic value of particular cortical changes in PDD development, namely more rapid cognitive decline. According to another study, atrophy in the frontal area, caudate nucleus, and SI could serve as predictors of dementia in PD^26^. Moreover, our colleagues recently reported a significant association between decreased fronto-striatal rsFC and a shorter duration of parkinsonism before MCI diagnosis in PD patients. The authors also found microstructural changes in the frontal white matter in PD-MCI patients with shorter disease duration, suggesting that the frontal area plays a pivotal role in deciding the rate of cognitive decline^27^. Our results also show an association between decreased rsFC in the frontal area and early cognitive decline in PD patients. However, future longitudinal studies with a larger study population with longer clinical follow-ups are required to confirm these findings.

Interestingly, the PD-MCI-SD group had decreased rsFC in the precuneus and bilateral angular gyri compared to the PD-MCI-LD group. These are key areas of the DMN, which has a pivotal role in cognitive function^28^. There have been growing evidence suggesting an association between cholinergic neurotransmission and DMN, showing cholinergic neuromodulation affect the brain activity during rest and task as well^29, 30^. A recent study also suggested that preserved cholinergic function in DMN as well as frontoparietal network might be a prerequisite for cognitive improvement after cholinergic treatment in PD patients^31^. In our study, compared to the PD-IC group, the direction of rsFC change in the PD-MCI-LD and the PD-MCI-SD groups was diverged, causing significant differences between the two groups. Possible different levels of burden in the cholinergic system between the PD-MCI groups might have caused this diverging change. Similar findings can be observed in Alzheimer’s disease: compensatory hyperconnectivity within DMN in MCI with less pathologic burden, followed by hypoconnectivity found in Alzheimer’s disease with more burden^32, 33^. It is unclear, however, whether we can apply this concept to rsFC between SI and precuneus and angular gyrus which normally have anti-correlation. Further study is required to unravel the association between the SI and DMN according to the degree of pathologic burden.

The present study has some limitations that need to be addressed. First, this is a cross-sectional study, so our results cannot be directly used to predict the rate of cognitive decline. Further prospective studies with regular follow-ups and comprehensive neuropsychological testing should be performed to validate our assumptions. Second, the onset timing of cognitive decline relies on subjective information; therefore, the temporal relationship between the onset of parkinsonism and cognitive decline is unavoidably subjective. More reliable and objective assessment methods need to be developed through future research. Third, the major nuclear cell group found in the SI is the NBM, but the SI is a neurochemically diverse area with both cholinergic and non-cholinergic projection neurons^34, 35^. Our results may be inevitably influenced by this heterogeneity of SI. Therefore, caution is needed when interpreting our results and future studies should be performed with more focus on the cholinergic neurons of SI. Fourth, because we performed seed-based rsFC analysis with only SI seed, we might have missed how other brain areas affect the rate of cognitive decline in PD patients. Therefore, a future study based on patterns of whole brain functional connectivity should be carried out to support our results and to determine which brain networks correlate with rapid cognitive decline in PD patients.

In conclusion, when using the bilateral SI as a seed, altered rsFC in the frontal and parietal areas might be relevant to cognitive dysfunction in PD patients. Furthermore, more functional burden in these areas, particularly decreased rsFC in the frontal areas might be associated with early-onset of MCI, suggesting that cholinergic deficits in the frontal areas might play an important role in the acceleration of cognitive decline and conversion to PD dementia.

## Methods

### Subjects

Patients were selected from a prospectively collected single-institution movement disorders and dementia outpatient clinic database between August 2011 and December 2015. Consecutive patients with drug-naïve de novo PD who underwent both MRI and neuropsychological tests within a 2-month interval were recruited. PD was diagnosed according to the clinical diagnostic criteria of the United Kingdom Parkinson’s Disease Society Brain Bank^36^.

Motor symptoms were assessed using the Unified Parkinson’s Disease Rating Scale Part III (UPDRS-III). Depressive symptoms were assessed using the self-rating Beck Depression Inventory^37^. We excluded patients with focal brain lesions, diffuse white matter (WM) hyperintensities outside the normal range, or multiple lacunar infarcts in the basal ganglia on MRI or other medical comorbidities that might account for cognitive dysfunction. All subjects completed MRI and [18F]-FP-CIT positron emission tomography (PET) imaging protocols. To ensure clinical diagnostic accuracy, only patients who exhibited decreased dopamine transporter uptake in the posterior putamen on a [18F]-FP-CIT-PET scan were included in this study.

Information about memory problems was gathered by interviews with the patients or caregivers. The cognitive status of PD patients was diagnosed by a group of two neurologists and one neuropsychologist in consensus, and diagnoses were based on a neuropsychological battery and physician-administered neurological examination.

The Seoul neuropsychological screening battery (SNSB), a detailed neuropsychological battery test standardized for the Korean population was used to evaluate cognitive performance^38, 39^. The SNSB is comprised of the forward and backward digit span test, Korean version of the Boston Naming Test (K-BNT)^40^, Rey complex figure test (RCFT, copying, immediate recall, 20-min delayed recall, and recognition), pentagon drawing test, Seoul Verbal Learning Test (SVLT, immediate recall, 20-min delayed recall, and recognition), phonemic and semantic Controlled Oral Word Association Test (COWAT), go-no-go test and contrasting program, and Stroop test (word and color reading of 112 items during a 2-min period). There were age-, sex-, and education-specific norms available for each test based on 447 healthy subjects. Patients were classified as abnormal when the scores of these tests were below the 16^th^ percentiles of the norms for the age-, sex-, and education-matched normal subjects. Except for the language domain, two neuropsychological tests were designated to represent each of the following four cognitive domains: 1. Attention (forward and backward digit span and Stroop color-word test); 2. Executive function (phonemic and semantic COWAT and clock drawing test); 3. Memory (SVLT and RCFT); 4. Visuospatial function (RCFT copy and pentagon drawing test); and 5. Language domain (only K-BNT).

According to the diagnostic criteria recommended by the Movement Disorder Society Task Force^41^, PD-MCI was diagnosed when at least two tests for each of the four domains other than the language domain were abnormal (level 2) or when at least two tests for each of the five domains were abnormal (level 1). Depending on the temporal relationship between the onset of parkinsonian motor symptoms and that of cognitive impairment, patients were divided into two groups of PD-MCI: PD-MCI < 1 year of parkinsonism before MCI diagnosis (PD-MCI-SD) and PD-MCI ≥ 1 year of parkinsonism before MCI diagnosis (PD-MCI-LD). Twenty-nine age- and sex-matched, drug-naïve de novo PD-IC patients were included as the control group.

### Standard protocol approvals, registrations, and patient consents

The study protocol was approved by the Yonsei University Severance Hospital ethical standards committee on human experimentation and was exempt from providing informed consent by the IRB due to its retrospective design. All experiments were performed in accordance with relevant guidelines and regulations.

### Image Acquisition

All participants underwent MR imaging with a 3-Tesla scanner (Intera Achieva, Philips Medical System, Best, the Netherlands) and a 32-channel head coil. Head motion was minimized with restraining foam pads offered by the manufacturer.

### Structural image acquisition

We used the 3-dimensional T1-turbo field echo sequence configured with the following acquisition parameters: axial acquisition with field of view = 220 mm; voxel size = 0.98 × 0.98 × 1.2 mm^3^; TE = 4.6 ms; TR = 9.6 ms; flip angle = 8°; slice gap = 0 mm; and total acquisition time = 5 min 29.3 s.

### Resting-state fMRI acquisition

The functional MR images were acquired by using a T2*-weighted single shot echo planar imaging sequence. For each subject, 165 axial volume scans were obtained with the following acquisition parameters: voxel size = 2.75 × 2.75 × 4.8 mm^3^; slice number = 31 (interleaved); matrix = 80 × 80; slice thickness = 4.8 mm; repetition time = 2000 ms; echo time = 30 ms; and field of view = 209 × 220 mm^2^. During functional MR imaging, subjects were instructed to remain awake with their eyes closed and to not move or focus on anything specific.

### Volumetric analysis of SI

Individual structural T1 images were processed using the Freesurfer software package version 5.3.0 (Massachusetts General Hospital, Harvard Medical School; http://surfer.net). Each subject’s structural images and the International Consortium for Brain Mapping (ICBM) 152 template were registered to a common spherical coordinate system^42, 43^. The SI region was manually drawn based on the ICBM 152 template by a radiologist (N.Y.S.; Supplementary Figure 3) and this was aligned to each subject’s structural volume with a nonlinear registration algorithm (Freesurfer’s mri cvs register and applyMorph). The delineation of the SI on MRI was based on a method reported previously by George and colleagues^44^. The volume was derived from three consecutive gapless 1 mm-thick slices on T1-weighted coronal images reformatted to be perpendicular to the anterior commissure (AC)-posterior commissure (PC) line. The three consecutive sections analyzed were at the level of the crossing of the AC, the level where the AC might be uncrossed, and the level of the emergence of the AC from the temporal lobe. The boundaries of the SI were as follows; the dorsal border was the ventral aspect of the globus pallidus, the ventral border was the base of the brain containing the anterior perforated space, the medial border was operationally defined by a vertical line extending from the ventrolateral border of the bed nucleus of the stria terminalis to the base of the brain, and the lateral border extended to the medial aspect of the putamen. The anatomical landmarks used to define the borders of the SI were applied to all three consecutive sections. For each subject, the structural volume was calculated and differences in SI volume between the three groups were analyzed. We also performed group comparisons between the collapsed PD-MCI group and the PD-IC group. Age, sex, years of education, and age at onset of parkinsonism were also included as covariates in ANCOVA.

### Analysis of rsFC

The rsfMRI data were analyzed using SPM8 (Wellcome Trust Centre for Neuroimaging, London, UK, www.fil.ion.ucl.ac.uk/spm/) implemented in MATLAB (The MathWorks, Inc. Natick, MA, USA). The preprocessing of rsfMRI Data was carried out using the Data Processing Assistant for Resting-State fMRI toolbox (http://www.restfmri.net) preprocessing pipeline. Preprocessing included slice timing, realignment (to the middle volume), coregistration, normalization (to the MNI space using T1 images), and smoothing (with a 4-mm full width at half maximum Gaussian kernel). Then, the preprocessed images were detrended and bandpass filtered (0.01~0.08 Hz). Nuisance covariates including head motion parameter, global mean signal, WM signal, and cerebrospinal fluid signal were regressed out. To perform a seed-based rsFC analysis, we first set the above-mentioned bilateral SI mask as a seed region (Supplementary Figure 3). Then, the correlation coefficient between the seed and remaining voxels in the whole brain was calculated. Individual *r* maps were normalized by Fisher’s *r*-to-*z* transformation, and the converted *z* maps were entered into group analysis. In the 2^nd^ level analysis, a pairwise two sample *t*-test was conducted to investigate differences in FC patterns between the collapsed PD-MCI group and the PD-IC group to see which brain regions were relevant to cognitive dysfunction in PD patients using the SPM8 toolbox. Afterwards, pairwise two sample *t*-tests were also performed to compare the PD-MCI-SD, PD-MCI-LD, and PD-IC groups with each other. Sex, years of education, age, age at onset of parkinsonism, and UPDRS III score were regressed out in the statistical test. Unless stated otherwise, the threshold for statistical analysis was set to *P* < 0.017 (Bonferroni corrected for three comparisons), based on the Monte Carlo Simulations with custom software implemented in MATLAB^45^.

### Statistical Analysis

The Kolmogorov–Smirnov test was used to determine normal distributions. Accordingly, quantitative data with normal distributions were presented as means ± standard deviations and were compared using ANOVA. Quantitative data without normal distributions were presented as medians with ranges and the Kruskal-Wallis test was used for analysis. The Chi-squared test or Fisher’s exact test was used to analyze qualitative data when appropriate. A post-hoc analysis was also performed using the Bonferroni-corrected Students’ *t*-test, Mann-Whitney *U* test, Chi-squared test, or Fisher’s exact test when appropriate with correction for multiple comparisons.

To test whether brain regions showing significant difference in the collapsed PD-MCI group compared to the PD-IC group were correlated with cognitive function, we performed a correlation analysis between those regions and neuropsychological test results. We first created ROI masks for the right frontal regions (40 voxels) showing decreased rsFC and the bilateral parietal regions (160 voxels) showing increased rsFC in the collapsed PD-MCI group, respectively. To assess which brain regions were associated with disease duration before MCI diagnosis, we created conjunction ROI masks by overlapping areas showing significantly different rsFC in PD-MCI-SD compared not only with PD-MCI-LD but also with PD-IC as there might be characteristic neural changes of PD-MCI-SD compared to PD-IC and PD-MCI-LD. Three frontal masks (right anterior frontal mask, 16 voxels; right superior frontal mask, 7 voxels; and left superior frontal mask, 15 voxels) were created for areas showing decreased rsFC in the PD-MCI-SD group compared with both the PD-MCI-LD and PD-IC groups, while the right parietal mask (3 voxels) was created for areas showing increased rsFC, which was excluded in the correlation analysis because the size of the area was too small. Then, mean *z* values extracted from the voxels within each ROI mask were correlated with neuropsychological test results or disease durations in all participants using Pearson’s or Spearman’s correlation coefficient, when appropriate.

Statistical analyses were performed using commercially available software (SPSS, version 21.0), and a two-tailed *P* value < 0.05 was considered significant.

## Electronic supplementary material

Supplementary Table & Figures
