# Supplementary material for: Early-onset mild cognitive impairment in Parkinson’s disease: Altered corticopetal cholinergic network
Source: Sci Rep. 2017 May 24;7:2381. doi: 10.1038/s41598-017-02420-w (PMC5443757; doi:10.1038/s41598-017-02420-w)

Title

Early-onset mild cognitive impairment in Parkinson's disease: Altered corticopetal cholinergic network

Injoong Kim, M.D.[1], Na-Young Shin, M.D.[2*], Yunjin Bak, M.A.[2], Phil Hyu Lee, M.D.[3], Seung-Koo Lee, M.D.[4], Soo Mee Lim, M.D.[5]

[1]Department of Radiology, Veterans Health Service Medical Center, Seoul, 05368, Korea

[2]Department of Radiology, College of Medicine, The Catholic University of Korea, Seoul, 06591, Korea

[3]Department of Neurology, Yonsei University College of Medicine, Seoul, 03722, Korea

[4]Department of Radiology, Yonsei University College of Medicine, Seoul, 03722, Korea

[5]Department of Radiology, Ewha Womans University School of Medicine, Seoul, 07985, Korea

*Corresponding author: Na-Young Shin, M.D. PhD.

Department of Radiology, College of Medicine, The Catholic University of Korea, Seoul, Korea

222 Banpo-daero, Seocho-gu,

Seoul 06591, Korea

TEL: 82-2-2558-1443

E-mail: nyshin@catholic.ac.kr

Supplementary Table 1. Group comparisons of rsFC between the bilateral SI and major brain regions in all group contrasts.

Contrast	Region	Side	MNI Coordinates			Maximum *t*	No. of voxels	*P* value
			x	y	z			
PD-MCI < PD-IC	Angular gyrus	R	45	-57	54	4.22	101	< 0.001
	Superior parietal gyrus	L	-18	-75	57	3.67	59	< 0.001
PD-MCI > PD-IC	Inferior orbitofrontal gyrus	R	42	39	-12	3.9	40	< 0.001
PD-MCI-SD < PD-IC	Superior frontal gyrus	R	24	54	6	5.37	81	< 0.001
	Middle frontal gyrus	R	30	18	42	4.97	40	< 0.001
	Medial superior frontal gyrus	R	12	33	45	4.53	33	< 0.001
	Middle frontal gyrus	L	-39	48	6	4.22	41	< 0.001
	Inferior frontal triangularis	L	-45	30	30	3.98	27	< 0.001
	Cerebellum	L	-9	-72	-30	3.88	25	< 0.001
	Thalamus	L	-6	-9	3	3.81	33	< 0.001
	Superior orbitofrontal gyrus	L	-15	63	-6	3.68	26	< 0.001
	Middle frontal gyrus	L	-36	15	57	3.55	25	< 0.001
PD-MCI-SD > PD-IC	Middle occipital gyrus	L	-18	-93	36	4.79	90	< 0.001
	Superior parietal gyrus	R	27	-63	60	4.38	141	< 0.001
	Precuneus	L	-12	-78	51	4.34	41	< 0.001
	Superior occipital gyrus	R	18	-87	33	4.15	84	< 0.001
	Inferior occipital gyrus	R	33	-93	-3	4.06	40	< 0.001
	Middle temporal gyrus	L	-63	-51	6	3.95	27	< 0.001
	Postcentral gyrus	R	51	-27	60	3.9	50	< 0.001
PD-MCI-LD < PD-IC	Inferior temporal gyrus	R	48	6	-45	4.31	28	< 0.001
	Inferior orbitofrontal gyrus	R	42	33	-12	3.6	37	< 0.001
PD-MCI-SD < PD-MCI-LD	Precuneus	L	-12	-51	36	4.86	147	< 0.001
	Middle frontal gyrus	L	-33	15	54	4.81	55	< 0.001
	Angular gyrus	R	51	-60	42	4.76	79	< 0.001
	Superior frontal gyrus	R	24	66	6	3.98	41	< 0.001
	Superior frontal gyrus	R	18	21	54	3.97	37	< 0.001
	Angular gyrus	L	-45	-60	39	3.95	32	< 0.001
PD-MCI-SD > PD-MCI-LD	Postcentral gyrus	R	48	-27	60	5.63	34	< 0.001

Supplementary Figure

Supplementary Figure 1.Mean rsFC with the bilateral SI seed extracted from areas showing significant differences between the PD-MCI-SD and PD-IC groups.

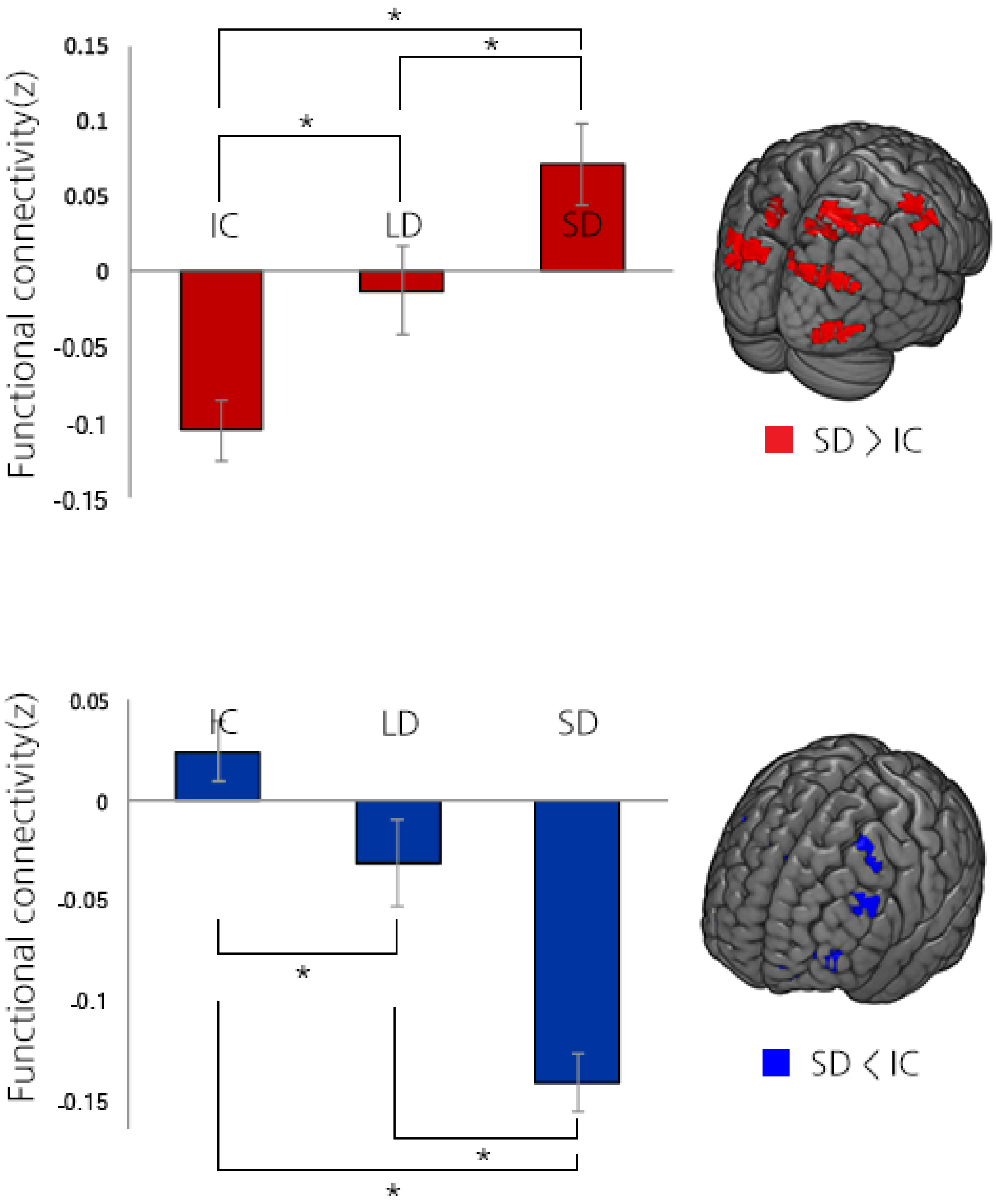

*: $P < 0.05$

Supplementary Figure 2. Mean rsFC with the bilateral SI seed extracted from areas showing significant differences between the PD-MCI-SD and PD-MCI-LD groups.

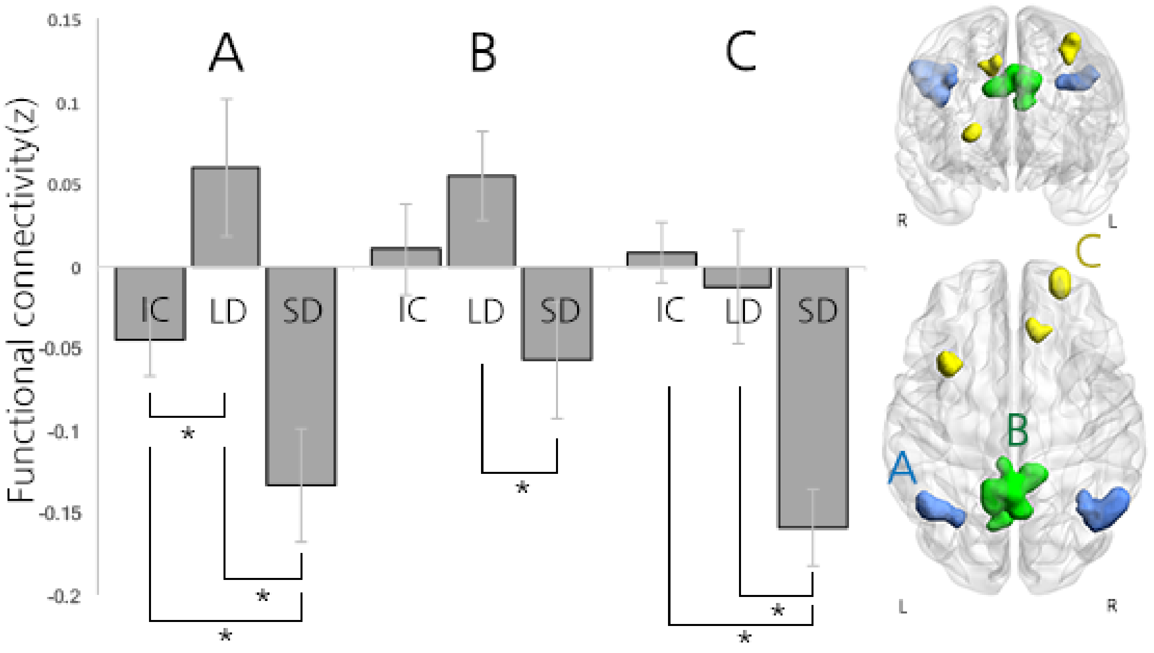

*: $P < 0.05$

Supplementary Figure 3. Bilateral SI seed. Coronal slice of the template image showing the location of the SI used in the seed-based rsFC analysis.

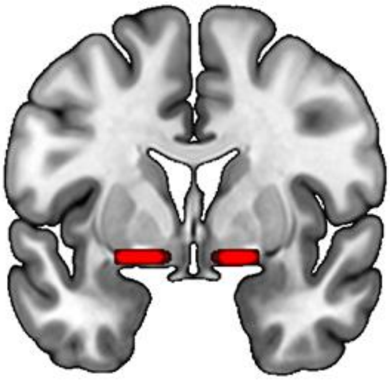

Supplement: Supplementary file 1 — Supplementary Table & Figures [file 41598_2017_2420_MOESM1_ESM.pdf]
